# Navigating the challenges: a case report on managing a complicated postpartum course in type 3 von Willebrand disease with alloantibodies

**DOI:** 10.1016/j.rpth.2024.102399

**Published:** 2024-04-03

**Authors:** Konrad van der Zwet, Karin P.M. van Galen, Annemiek C.C. Evers, Kathelijn Fischer, Roger E.G. Schutgens, Lize F.D. van Vulpen

**Affiliations:** 1 Center for Benign Haematology, Thrombosis and Haemostasis, Van Creveldkliniek, University Medical Center Utrecht, Utrecht University, Utrecht, The Netherlands; 2 Obstetrics and Gynaecology, University Medical Center Utrecht, Utrecht, The Netherlands

**Keywords:** alloantibodies, emicizumab, postpartum hemorrhage, von Willebrand disease

## Abstract

**Background:**

Von Willebrand disease (VWD) type 3 is characterized by a complete deficiency of von Willebrand factor (VWF), resulting in a severe bleeding phenotype. Treatment often requires administration of VWF concentrates/factor (F)VIII. However, the development of alloantibodies is a rare complication, resulting in ineffective recovery and allergic reactions. Emicizumab, a bispecific antibody mimicking FVIII function, has emerged as a potential alternative, with promising results reported in several case reports.

**Key Clinical Question:**

Description of multiple approaches to control highly severe postpartum hemorrhage in type 3 VWD with alloantibodies, including off-label use of emicizumab.

**Clinical Approach:**

Here we present a 28-year-old patient with type 3 VWD and alloantibodies, known to have arthropathy of the right elbow. Previous immune tolerance induction was unsuccessful. Despite receiving negative pregnancy advice during preconception counseling, the patient became pregnant. Delivery was induced at 38 4/7 weeks with prostaglandin, and recombinant FVIIa (rFVIIa) was administered every 2 hours. Despite administration of rFVIIa, bleeding persisted, requiring manual placental removal and insertion of a Bakri balloon. Since bleeding persisted, plasma-derived VWF was administered with an initial excellent recovery and successful embolization of the uterine artery. Twelve days postpartum, she developed endometritis and recurrent vaginal bleeding treated with antibiotics, rFVIIa every 2 hours, and multiple erythrocyte transfusions. Plasma-derived VWF was administered but was complicated by anaphylaxis and no recovery. Due to persistent vaginal bleeding, reembolization of uterine arteries was performed and off-label emicizumab was initiated. Twenty-nine days postpartum, she developed septic shock requiring an abdominal hysterectomy, again complicated by severe bleeding necessitating direct intraabdominal packing after rFVIIa. A computed tomography scan 9 days postsurgery revealed thrombosis in the left iliac vein and asymptomatic pulmonary embolisms. rFVIIa was stopped and prophylactic low-molecular-weight heparin was started. The patient was discharged 2 months after delivery on low-dose low-molecular-weight heparin, emicizumab, and antibiotics for an intra-abdominal abscess. During 2.5 years of emicizumab prophylaxis, she has had no rebleeding in her arthropathic right elbow.

**Conclusion:**

The current case emphasizes the postpartum clinical challenges of patients with type 3 VWD and alloantibodies. It underscores the potential role of emicizumab in maintaining hemostatic control.

## Essentials

• Von Willebrand disease (VWD) type 3 is a complete deficiency of the von Willebrand factor.
• Alloantibodies in VWD type 3 are rare, causing impaired recovery on von Willebrand factor/factor VIII concentrates.
• Managing delivery in VWD type 3 with alloantibodies is a unique and complex clinical challenge.
• Emicizumab offers a potential new role in the treatment of severe postpartum hemorrhage.

## 1 Introduction

Von Willebrand disease (VWD) type 3 is characterized by the complete deficiency of von Willebrand factor (VWF) [1]. The prevalence of VWD type 3 varies worldwide but is estimated at 0.1 to 5.3 cases per million population [2].

Patients with VWD type 3 have a severe bleeding phenotype manifesting in mucocutaneous bleeding and/or hemarthroses, often requiring replacement therapy with VWF concentrates/factor (F)VIII. While replacement is mostly effective in preventing bleeding episodes, 5% to 10% of patients with type 3 VWD develop alloantibodies to VWF/FVIII concentrates, resulting in ineffective recovery on VWF/FVIII concentrates, and/or administration of these concentrates may be complicated by allergic reactions [2,3]. Currently, patients are mainly treated with high doses of recombinant FVII (rFVIIa) during bleeding episodes and invasive medical procedures [1,2].

Emicizumab is a bispecific antibody that partially mimics the function of coagulation FVIII and has been approved by the Food and Drug Administration and European Medicines Agency for patients with severe and moderate hemophilia A [4]. However, off-label use of emicizumab has been reported in several case reports of patients with type 3 VWD and alloantibodies [[5], [6], [7]].

Here we present a case outlining the management of multiple agents, including the first case using off-label emicizumab to maintain hemostatic control in a patient with type 3 VWD and alloantibodies who experienced severe and prolonged bleeding after vaginal delivery.

## 2 Case Report

We report the case history of a 28-year-old patient with type 3 VWD and alloantibodies to VWF and FVIII (anti-FVIII: 1.5 Bethesda Units). She was diagnosed with type 3 VWD during infancy (homozygous deletion of VWF gene). Her family history reported parental consanguinity (first cousins). At the age of 3.0 years, the patient developed alloantibodies and immune tolerance induction was initiated. Unfortunately, the immune tolerance induction was unsuccessful and complicated by anaphylaxis. Throughout her childhood and adolescence, she reported recurrent musculoskeletal bleeding that started at the age of 4 years. Later, she experienced repeated nose bleeds and suffered from heavy menstrual bleeding, which was controlled with oral contraceptive and tranexamic acid. Due to recurrent joint bleeding in her right elbow, she developed arthropathy, necessitating resection of osteophytes in the right olecranon and radiosynovectomy.

Despite extensive preconception counseling about the risks around labor and negative pregnancy advice, the patient became pregnant. During the second trimester, she caught a mild COVID-19 infection, but no other complications occurred. Delivery was induced at 38 4/7 weeks using prostaglandin. During active labor, rFVIIa (90 μg/kg) was administered every 2 hours and 1 g of tranexamic acid was administered after clamping of the umbilical cord and continued every 8 hours. A healthy female neonate was delivered with a mildly decreased VWF activity of 47% and FVIII activity of 82%. Oxytocin (5 IU) was administered for placental retention. Despite frequent administration of rFVIIa, vaginal bleeding persisted, resulting in a significant decrease in hemoglobin levels from 12.7 g/dL to 6.3 g/dL. Consequently, manual placental removal was required, followed by the insertion of a Bakri balloon, along with transfusion of erythrocytes, platelets, and plasma and continuation of rFVIIa (90 μg/kg) and tranexamic acid 3dd 1 g. A total of 3 L blood loss was observed in the first 24 hours. Since vaginal bleeding persisted, plasma-derived VWF (150 IU/kg) was administered with corticosteroids and clemastine. Initially, this resulted in an excellent recovery: the VWF activity increased from <0.5% to 206% and FVIII from 1.9% to 194%. However, continuous bleeding necessitated embolization of both uterine arteries 48 hours after delivery. To prevent rebleeding, another infusion of plasma-derived VWF (25 IU/kg) was administered. However, this second infusion of VWF resulted in lacked recovery.

Due to the persistent high FVIII levels and risk of thrombosis, rFVIIa was stopped 3 days after initiation. No rebleed was observed during hospital admission, and the patient was discharged 8 days postpartum.

Twelve days postpartum, the patient was readmitted for endometritis. Although no vaginal bleeding was observed upon admission, rebleeding occurred 5 days after admission, requiring rFVIIa (90 μg/kg) administration every 3 hours. Despite repeated rFVIIa administration and multiple erythrocyte transfusions, vaginal bleeding persisted, with a significant decrease in hemoglobin to 7.1 g/dL. Another infusion of plasma-derived VWF (100 IU/kg) was administered with corticosteroids and clemastine but was complicated by an anaphylactic reaction and no recovery; therefore, reembolization was performed. Off-label emicizumab was started to prevent rebleeding after obtaining informed consent. A loading dose of 6 mg/kg on day 1 and 3 mg/kg on day 2 was given, followed by 3 mg/kg every other week (150 mg) from the second week onward, according to Tiede et al. [8]. The reembolization was successful, and rFVIIa could be tapered and discontinued after 14 days after admission. Unfortunately, she developed septic shock 29 days postpartum despite broad-spectrum antibiotics. Abdominal hysterectomy was required to control the infection. The surgery was performed after a loading dose of rFVIIa (200 μg/kg), but despite this being complicated by a severe bleed requiring direct intraabdominal packing, rFVIIa (90 μg/kg) every 2 hours and recombinant VWF (200 IU/kg) were administered. However, no recovery was observed (FVIII 7%, VWF antigen: 2.2, VWF activity: <0.5%).

While bleeding decreased on a follow-up CT scan 9 days after surgery, thrombosis of the left iliac vein and asymptomatic pulmonary embolisms were detected as coincidental findings. rFVIIa was discontinued, and 2500 E low-molecular-weight heparin twice daily was started. During admission, another episode of vaginal bleeding was reported. A third embolization was performed on the left internal iliac artery after platelet transfusion, with no further complications.

Ultimately, 2 months after delivery, the patient was discharged with a regimen of low-dose (50 IU/kg) low-molecular-weight heparin once daily for 6 weeks, emicizumab, and antibiotics for treatment of an intraabdominal abscess. In total, 42 erythrocytes, 27 plasma, and 23 platelet transfusions had been administered during both hospital admissions; see the Figure for details. She continued emicizumab prophylaxis off-label every 2 weeks with overall good bleeding control. She was readmitted twice, requiring rFVIIa administration due to a spontaneous intra-articular bleed in her right shoulder and an infected hematoma after ovulatory bleed in her pelvis. Since the start of emicizumab prophylaxis, no bleeds have reoccurred in her arthropathic right elbow. The annual bleeding rate improved from 3.93 before emicizumab to 1.25 during emicizumab and her annual joint bleeding rate improved from 2.86 to 0.25.

**Figure:**
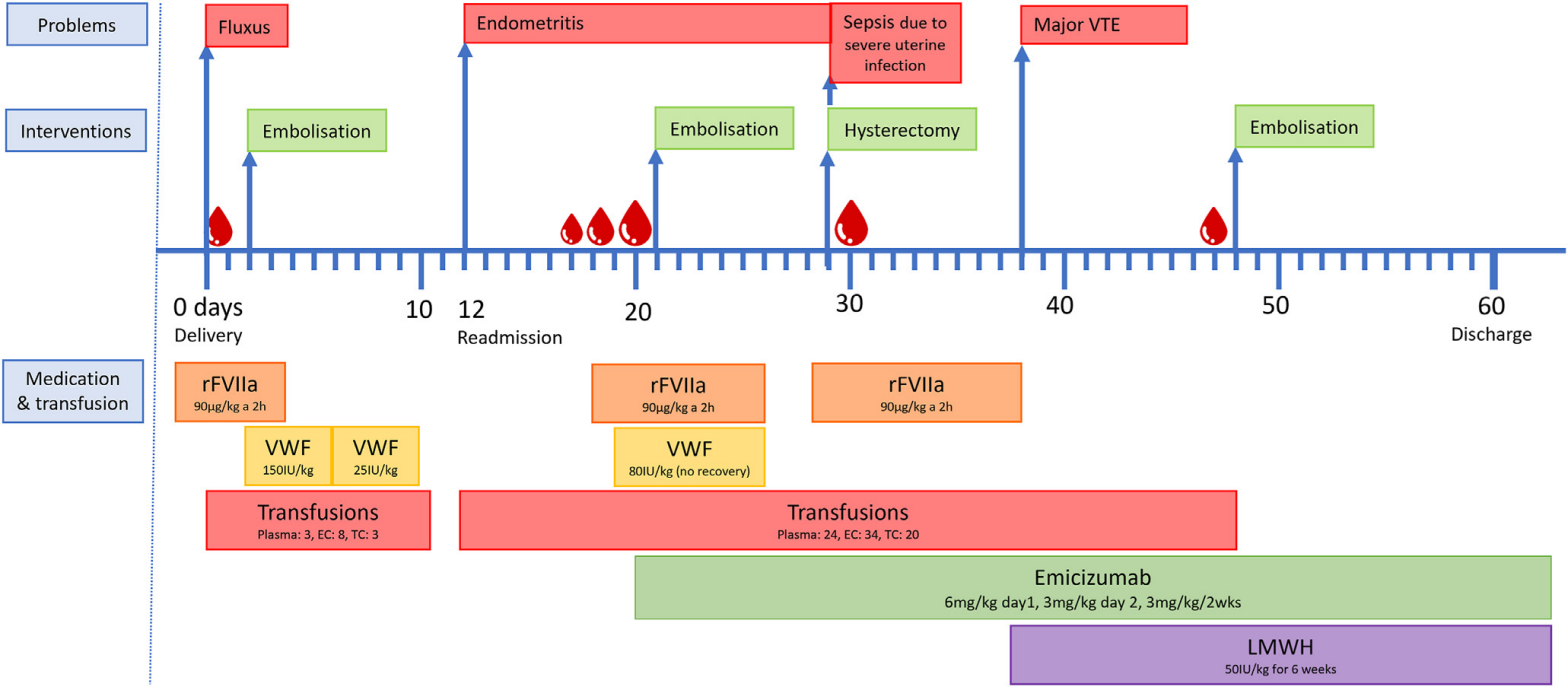
Case report timeline. EC, erythrocyte concentrate; LMWH, low-molecular-weight heparin; rFVIIa, recombinant factor FVII; TC, thrombocyte concentrate; VTE, venous thromboembolism; VWF, von Willebrand factor.

## 3 Discussion

This case illustrates the high-risk situation of vaginal delivery in a patient with VWD type 3 in the presence of alloantibodies. This case was complicated by life-threatening bleeding postpartum, requiring frequent coagulation correction and embolization. In addition, the patient developed severe sepsis due to endometritis, which eventually resulted in a hysterectomy with again heavy blood loss, requiring again intensive coagulation correction and embolization. Finally, multiple risk factors (eg, surgery, immobility, and intensive coagulation corrective therapy) resulted in the third severe complication: venous thrombosis, necessitating the initiation of therapeutic anticoagulation with the risk of bleeding.

The management of delivery in patients with type 3 VWD and alloantibodies has been described in limited cases with varying approaches; see the Table [[9], [10], [11]]. Scott et al. (2017) [9] described the case of a 29-year-old patient during labor; they administered rFVIIa (270 μg/kg) every 2 hours, alongside 2 units of platelets 4-hourly and tranexamic acid (1 g) 6-hourly until hemostasis. Due to a failure in progress of the delivery, the patient required a cesarean section. A total of 1800 mL blood loss was observed secondary to uterine atony, requiring insertion of a balloon tamponade and uterotonics. rFVIIa was discontinued and platelet support 4 days after delivery and tranexamic acid was given for 7 days.

**Table:** Summary of case reports on delivery management in patients with von Willebrand disease type 3 and alloantibodies.

Authors	Patient characteristic	Initial treatment	Complications	Total blood loss (mL)
Scott et al. (2017) [9]	29 y/GA + 39 wk, spontaneous labor	• FVIIa every 2 h (270 μg/kg) • 2 packs of platelets 4 h • Tranexamic acid	Cesarean section due to failure in progress, followed by secondary uterine atony requiring additional insertion of balloon tamponade and uterotonics.	1800
Boyer-Neuman et al. (2003) [10]	29 y/cesarean section	• rFVIII bolus 100 IU/kg, followed by continuous infusion 35 IU/kg	+15 d postpartum uterine bleeding, as a consequence of refractory high dose of rFVIII infusion requiring a switch to rFVIIa (270 μg/kg) +19 d postpartum venous thrombosis discontinuation of rFVIIa, followed by uterine rebleeding, requiring multiple EC transfusions. Ultimately a vena cava filter was placed in combination with an embolization of the uterine arteries.	NA
Martín-Salces et al. (2012) [11]	30 y/cesarean section	• FVIII/VWF bolus (100 IU/kg), followed by continuous infusion of FVIII/VWF (10 IU/kg/h) • Tranexamic acid	+2 h after caesarian section development of uterine bleeding, requiring EC and TC transfusions. +5 d postpartum development of intramural uterine hematoma, requiring additional bolus of FVIII/VWF (60 IU/kg) and continuous infusion of FVIII/VWF (8 IU/kg/h) for 4 d	NA

EC, erythrocyte concentrate; FVIII, factor VIII; GA, gestational age; NA, not applicable; rFVIII, recombinant FVIII; TC, thrombocyte concentrate; VWF, von Willebrand factor.

Boyer-Neumann et al. (2003) [10] reported a case of a 29-year-old patient who received continuous rFVIII infusion (35 IU/kg/h) prior to a cesarean section; no bleeding was observed during the cesarean section. However, the patient became refractory to high dose rFVIII and developed uterine bleeding 15 days postpartum, requiring rFVIIa infusion (270 μg/kg) every 4 hours. Although the uterine bleeding was treated effectively, the patient developed an ilio-femoral venous thrombosis, and rFVIIa was discontinued. Subsequently, rebleeding reoccurred, requiring multiple erythrocyte transfusions. A vena cava filter was placed, followed by embolization of the uterine arteries, both performed under single administration of rFVIIa. The embolization was successful in stopping the uterine bleeding; no further bleeding was observed [10].

Thirdly, Martin-Salces (2012) [11] reported a case of 30-year-old patient who received a loading dose of VWF/FVIII concentrate (100 IU/kg) prior to the cesarean section, followed by continuous infusion of VWF/FVIII concentrate (10 IU/kg/h). Although no bleeding was observed during the cesarean section, the patient developed uterine bleeding 2 hours after surgery, requiring 2 erythrocyte concentrates and platelet transfusions. Subsequently, admission was complicated by an intramural uterine hematoma requiring erythrocyte transfusion; a bolus of VWF concentrate (60 IU/kg) was administered, followed by continuous infusion of VWF/FVIII concentrate (8 IU/kg/h). No further bleeding complications were observed [11].

These 3 case reports complement our case describing life-threatening situations in the delivery of patients with VWD type 3 and alloantibodies. On the one hand, it is important to strive for good hemostasis and, on the other, to be vigilant for thrombosis. In contrast to these case reports, our patient had a vaginal delivery, which has shown to have a lower incidence of postpartum hemorrhage in patients with VWD [12]. However, the safest mode of delivery in such complex VWD cases with inhibitors is unknown.

To our knowledge, this is the first patient with type 3 VWD and alloantibodies who received emicizumab when experiencing postpartum hemorrhage. Several case reports have described the role of emicizumab prophylaxis in children with type 3 VWD with recurrent hemarthroses and progressive arthropathy, maintaining excellent bleeding control after initiation of emicizumab.

The good bleeding control with emicizumab prophylaxis suggests that correction of FVIII deficiency with a mimicking FVIII-antibody (emicizumab) in type 3 VWD improves the bleeding phenotype in these severely affected patients, despite having low VWD activity [[5], [6], [7]]. These case reports complement previous literature indicating that joint bleeds in VWD patients are associated with low FVIII levels [13]. The use of emicizumab prophylaxis and the concomitant indirect correction of the FVIII deficiency could thus potentially reduce joint bleeding and/or rebleeds in patients with type 3 VWD [14]. However, this has only been demonstrated in 3 case reports thus far. Furthermore, these case reports were conducted in children, which makes comparison with our case difficult [[5], [6], [7]]. Also, it appeared that emicizumab prophylaxis could not prevent vaginal rebleeds and operative bleeding in our patient. Whether its initiation played a role in the final control of her postpartum bleeding complications is uncertain. Nevertheless, during 2.5 years of emicizumab prophylaxis following the near-fatal delivery, no rebleeding was observed in her arthropathic right elbow. An ongoing clinical trial is currently evaluating the safety and efficacy in children and adults with severe type 3 VWD (ClinicalTrial.Gov identifier: NCT05500807).

## 4 Conclusion

Managing delivery and the postpartum course after vaginal delivery in a patient with type 3 VWD and alloantibodies is a significant clinical challenge. A negative pregnancy advice in such cases is justified to preserve a woman’s health. In addition to optimizing vaginal bleeding with intensive coagulation correction, patients face additional risk factors, including infection and surgery, making them susceptible to thrombotic events. Emicizumab represents a novel therapeutic alternative for patients with type 3 VWD and alloantibodies that can be administered during episodes of severe postpartum hemorrhage and to prevent recurrent joint bleeding in arthropathic joints.
